# Does early identification of work-related stress, combined with feedback at GP-consultation, prevent sick leave in the following 12 months? a randomized controlled trial in primary health care

**DOI:** 10.1186/s12889-019-7452-3

**Published:** 2019-08-14

**Authors:** K. Holmgren, G. Hensing, U. Bültmann, E. Hadzibajramovic, M. E. H. Larsson

**Affiliations:** 1 Department of Health and Rehabilitation, Institute of Neuroscience and Physiology, The Sahlgrenska Academy at the University of Gothenburg, Gothenburg, Sweden; 2 Section for Epidemiology and Social Medicine, Institute of Medicine, The Sahlgrenska Academy at the University of Gothenburg, Gothenburg, Sweden; 3 Department of Health Sciences, Community and Occupational Medicine, University of Groningen, University Medical Center Groningen, Groningen, The Netherlands; 4 Institute of Stress Medicine, Region Västra Götaland, Gothenburg, Sweden; 5 Health Metrics, Department of Public Health and Community Medicine, Institute of Medicine, Sahlgrenska Academy, University of Gothenburg, Gothenburg, Sweden; 6 Närhälsan Research and Development, Primary Health Care, Region Västra Götaland, Gothenburg, Sweden

**Keywords:** Psychosocial work factors, Work stress questionnaire (WSQ), Intervention, Organizational climate, Work commitment, Primary health care

## Abstract

**Background:**

Experiencing work-related stress constitutes an obvious risk for becoming sick-listed. In primary health care, no established method to early identify, advise and treat people with work-related stress exists. The aim was to evaluate if the use of the Work Stress Questionnaire (WSQ) brief intervention, including feedback from the general practitioner (GP), had an impact on the level of sickness absence.

**Method/design:**

In total 271 (intervention group, *n* = 132, control group, *n* = 139) non-sick-listed employed women and men, aged 18 to 64 years, who had mental and physical health complaints and sought care at primary health care centers participated in this two-armed randomized controlled trial. The main outcomes were the number of registered sick leave days and episodes, and time to first sick leave during the 12-months follow-up. The intervention included early identification of work-related stress by the WSQ, GP awareness supported by a brief training session, patients’ self-reflection by WSQ completion, GP feedback at consultation, and initiation of preventive measures.

**Results:**

The mean days registered for the WSQ intervention group and the control group were 39 and 45 gross days respectively, and 31 and 39 net days respectively (ns). No statistical significant difference for the number of sick leave episodes or time to first day of sick leave episode were found between the groups.

**Conclusions:**

The WSQ brief intervention combined with feedback and suggestions of measures at patient–GP-consultation was not proven effective in preventing sick leave in the following 12 months compared to treatment as usual. More research is needed on methods to early identify, advise and treat people with work-related stress in primary health care, and on how and when GPs and other professionals in primary health care can be trained to understand this risk of sick leave due to work-related stress, on how to prevent it, and on how to advise and treat employees at risk.

**Trial registration:**

ClinicalTrials.gov. Identifier: NCT02480855. Registered 20 May 2015.

## Background

Work-related stress constitutes a risk to the development of mental health problems such as depressive symptoms [1, 2], mood and anxiety disorders [3], and musculoskeletal disorders [4]. In a working age population seeking primary health care due to physical and mental symptoms almost 60% reported a moderate to high level of perceived overall stress [5]. Among employed non-sick-listed women and men seeking primary health care, approximately one third reported high perceived stress due to poor organizational climate and high work commitment [6]. Perceiving work-related stress constitutes a risk for people to become sick-listed [6–9]. To early identify, advise and treat people with work-related stress in primary health care might be a promising step in preventing sickness absence.

People with different kinds of physical and mental symptoms often visit the primary health care [10–12], even long before sick leave [6, 13]. Screening for work-related stress seems vital to prevent long-term sick leave and disability [14–16]. However, to date the screening instruments are mainly directed at patients with specific diagnoses, such as low-back pain, musculoskeletal complaints [15, 17] or mental health problems [18]. In the primary health care setting, patients present with a large variety of diagnoses [19]. The Work Stress Questionnaire (WSQ) was developed in the primary health care context from the perspective of the sick-listed patient. The WSQ was designed to early identify people with work-related stress at risk for sick leave [6, 13, 20], and takes both work-related factors and personal characteristics into account.

Furthermore, the patients’ working conditions are rarely touched upon at primary health care consultations [21–23]. The general practitioners (GPs) report little knowledge of the influence of psychosocial and organizational work factors on health and risk for sick leave, [22, 24], and they often do not talk to their patients about work-related issues [21, 23]. There is an obvious need to raise the GPs’ awareness of the risk for future sickness absence due to work-related stress, and to support GPs to early identify work-related stress to prevent sickness absence.

**Fig. 1:**
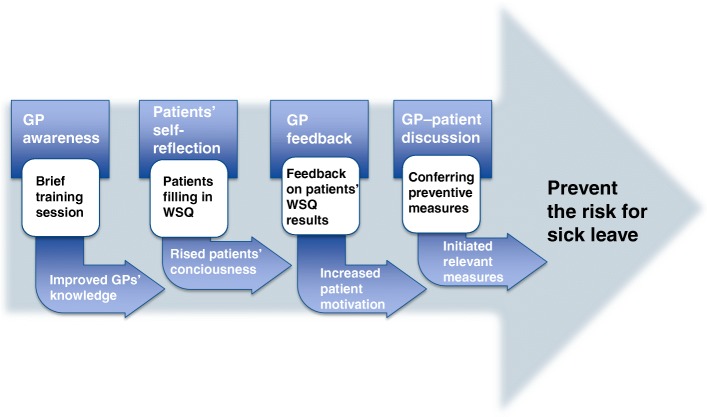
The WSQ brief intervention main components, TIDAS in New Ways

Both pragmatic and theoretical considerations guided the design of the WSQ brief intervention in the primary health care setting. The intervention consisted of four main components: 1) GP awareness supported by a brief training session, 2) self-reflection of patients upon completion of the WSQ, 3) patient motivation to address their work situation reinforced by GP feedback on the WSQ results, and 4) GP–patients discussions and initiation of preventive measures (Fig. 1). Through GP brief training sessions, we expected to improve GP knowledge on work-related stress. Further, we hypothesized that the first three components of the intervention would constitute a basis for fruitful discussions, at the GP-patient consultation, on relevant measures preventing the risk for sick leave (Fig. 1).

## Methods

The objective of this study was to evaluate the effect of the WSQ brief intervention including GP feedback, in the primary health care setting, compared to treatment as usual. Main effect measures were 1) the number of registered sick leave days (i.e. 14 days or more), 2) the number of absence episodes during 12 months after inclusion, and 3) time to first sick leave during 12-months follow-up.

### Study design

The project ‘Early identification of persons at risk for sick leave due to work-related stress’ named TIDAS was designed as a two-armed randomized controlled trial (RCT) comprising an intervention group and a control group with a 12-months follow-up period [25]. TIDAS is part of the research program New Ways – mental health at work, aimed at identification, treatment and support of persons with common mental disorders (CMD) to remain in work. For the present study, the primary outcome was sickness absence derived from registers of the Swedish Social Insurance Agency (SSIA), and was designed in accordance with CONSORT recommendations [26]. TIDAS is presented in detail in a study protocol [25].

The study took place in the Region Västra Götaland, Sweden. Seven PHCC located in urban and rural areas in and around Gothenburg took part and baseline data was collected over a period of 4–12 weeks per PHCC. The recruitment was completed between May 2015 and January 2016.

### Randomization and recruitment

**Fig. 2:**
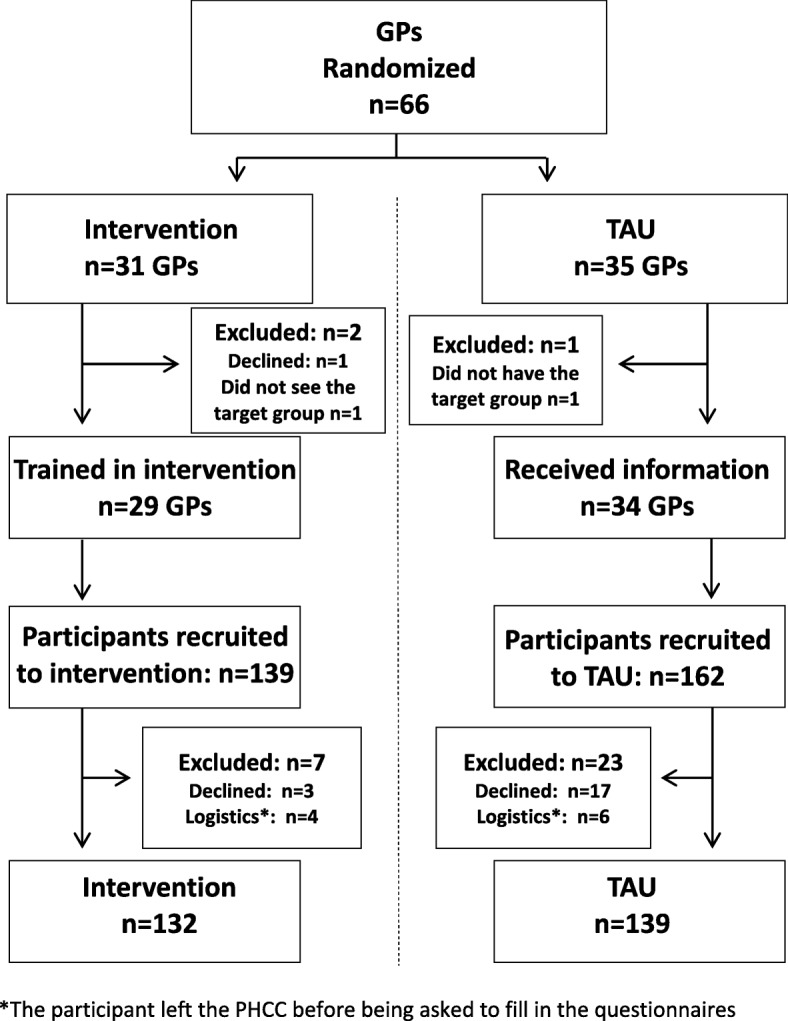
Flowchart of enrolment, allocation and baseline, TIDAS in New Ways

At the participating PHCCs GPs and residents were randomized to intervention GPs (*n* = 31) and control GPs (*n* = 35). The advantages for this were considered twofold: to reduce the risk for differences in socioeconomic factors between participating patients in intervention and controls, and to promote a high participation by engaging the whole PHCC to recruit both to intervention and control groups at the same time [27]. Folded slips of paper with the written names were mixed in a non-transparent bowl and subsequently drawn, one at a time, to either intervention or control group by colleagues not involved in the RCT. A research assistant was placed at the PHCC, who consecutively recruited patients with the required qualifications. The research assistant, with help from the reception-personnel, identified potential participants based on the registered reason for the consultation. Included were non-sick-listed employed patients aged 18 to 64 years who attended the PHCCs for mental and/or physical health complaints. Patients who had been off work due to sickness for a total of 7 days or more during the last month because of sickness, and those with a full or part-time disability pension were also excluded (Fig. 2).

### Intervention and control

The WSQ brief intervention comprised a) a 2 h GP training in the use of the WSQ and gaining knowledge on the relation between work-related stress and health, b) giving information to the GPs on access to preventive measures by the services of the primary health care specialists and occupational healthcare, c) the completion of the WSQ by participants before the GP consultation, d) the computation and analysis of the WSQ by a research assistant and provision of the result to the GP before consultation, e) the provision of feedback by the GP to the participant on WSQ results at the consultation, and d) a discussion between GP and participant and suggestions on measures to be taken. The intervention was not expected to prolong the GP-consultation, but to be done within the ordinary time limit.

The previous developed self-administered WSQ [20] includes 21 questions grouped in the following four categories: perceived stress owing to *indistinct organization and conflicts* and to *individual demands and commitment, and influence at work* and *work interference with leisure time.* The reliability and face validity of the WSQ has been tested and found satisfying [20].

The control group received treatment as usual at the patient–GP consultation, which can consist of medical investigation, diagnostics, treatment, and discussion about preventive and rehabilitating measures. Following that, the patients filled in the WSQ and were asked for background characteristics. Their GPs were not informed if the patients belonged to the study or not, and were to carry on with their usual consultations.

### Sample size

To identify at least 15% difference between the intervention and the control group when it comes to the number of sick leave days from the SSIA, (i.e. > 14 days or more) during 12 months after inclusion, the power calculation (with a two-sided test, statistical significance of *p* < 0.05 and 80% power) showed that 135 participants were needed in each group [25].

### Blinding

Blinding the research assistant, the participants or the GPs was not possible as all participants received the study information by the research assistant, and the intervention GPs received information and training before the study started. The control GPs received information about the study but no training and provided care as usual to their patients.

### Primary outcome measures

The primary outcome measures were the number of registered sick leave days and sick leave episodes. Data was procured from the SSIA’s Micro Database for Analyzing Social insurance (MiDAS): registered gross (number of sick-days, irrespective of extent of sick leave) and net (number of sick-days converted into whole days) sick-days, and the number of sick leave episodes, sickness and activity compensation during the 12 months following baseline.

The so-called rehabilitation chain, introduced by the Swedish government in 2008, is a well-defined process to promote return to work, in the employer has to introduce new measures when the sick leave has reached the fixed critical point of 90 days [28]. Therefore, sick leave days were divided into ≤90 registered sick-days and > 90 days registered sick-days.

### Data management and statistical analyses

Differences between the intervention and control groups concerning baseline characteristics and number of registered sick leave days and sick leave episodes were tested with chi-square test (dichotomous variables) or Mann-Whitney test (categorical variables). The 95% confidence interval (CI) for difference in proportion of high work-related stress assessed by the WSQ was calculated for the intervention and control group [29]. Kaplan-Meier survival curves were calculated for time to first day of sick leave episode for the two groups and the differences were tested with the log-rank test [29]. All analyses were performed using SPSS 22.0.

## Results

### Study population

The study population comprised 271 participants, *n* = 132 in the WSQ brief intervention group and *n* = 139 in the control group (Fig. 2). There were no statistically significant differences between the study population and those who declined participation or those who were excluded concerning gender and age [25]. The majority of participants were women in both the WSQ brief intervention group (67%) and the control group (70%) (Table 1).

**Table 1:** Baseline characteristics of the study population, *n* = 271. TIDAS in New Ways

	WSQ intervention group, *n* = 132	Control group, *n* = 139
	n^a^ (%)	n^a^ (%)
Gender		
Female	88 (67)	97 (70)
Age groups*		
19–30 years	21 (16)	26 (19)
31–50 years	58 (44)	76 (54)
51–64 years	53 (40)	37 (27)
Birthplace		
Nordic countries	122 (93)	125 (90)
Other	9 (7)	14 (10)
Educational level		
Compulsory schooling	13 (10)	15 (11)
Secondary school	61 (46)	59 (42)
University or higher	57 (44)	65 (47)
Occupational class		
Skilled/unskilled manual	49 (37)	58 (42)
Medium/low non-manual	60 (46)	56 (41)
High-level non-manual	23 (17)	24 (17)
Employer		
Private	61 (46)	68 (49)
Public	66 (50)	61 (44)
Self-employed	5 (4)	9 (7)
WSQ^b^		
Indistinct organization & conflicts	28 (21)	26 (19)
High work commitment	63 (48)	61 (44)
Low influence at work	54 (41)	54 (39)
Work to leisure time interference	54 (41)	55 (40)

^a^Dispersed numbers of participants are owing to internal missing data

^b^Work Stress Questionnaire

*Statistically significant difference, Mann-Whitney U test, *p* = 0.042

The baseline prevalence of overall perceived stress due to indistinct organization and conflicts was 21% in the intervention group and 19% in the control group, and 48 and 44% respectively for overall perceived stress due to high work commitment (Table 1).

Registered sick leave was largely skewed. During the following 12 months following baseline, the median was 0 days of registered sick leave, regardless of counting gross days or net days in both groups. The mean days registered for the WSQ intervention group and the control group were 39 and 45 gross days respectively, and 31 and 39 net days respectively (ns). These differences were not found to be statistically significant, nor were the differences between the groups at the 3, and 6 months follow-ups (Table 2).

**Table 2:** Comparisons of registered sick leave days between the WSQ intervention (*n* = 132) and the control group (*n* = 139)

Time period	WSQ intervention group, *n* = 132				Control group, *n* = 139				*p*-value
During	mean	sd	median	Q1;Q3	mean	sd	median	Q1;Q3	
3 months following baseline, gross days	11.39	25.12	0	0;3.75	11.24	24.90	0	0;0	0.667
3 months following baseline, net days	9.07	20.96	0	0;3.75	9.58	22.60	0	0;0	0.685
6 months following baseline, gross days	21.98	49.13	0	0;10.75	22.97	48.90	0	0;10	0.869
6 months following baseline, net days	16.98	39.71	0	0;7.75	18.82	41.77	0	0;10	0.930
12 months following baseline, gross days	38.98	86.37	0	0;22.5	45.14	84.65	0	0;60	0.626
12 months following baseline, net days	30.60	71.20	0	0;13.75	38.99	68.86	0	0;46	0.612

Mann-Whitney Test, *sd* standard deviation, Q1;Q3 = first and third quartile

At 12 month follow-up, no statistically significant differences between the intervention and the control group were found with regard to registered sick leave of ≤90 days (86% *n* = 114 and 79% *n* = 110, respectively) or > 90 days (14% *n* = 18 and 21% *n* = 29, respectively).

The number of sick leave episodes is presented in Table 3. No statistical significant difference was found between the intervention and control group.

**Table 3:** Comparisons of the number of registered sick leave episodes between the WSQ intervention (n = 132) and the control group (n = 139)

Number of sick leave episodes	WSQ intervention group, *n* = 132 n (%)	Control group, *n* = 139 n (%)
0	83 (62.9)	87 (62.6)
1	39 (29.5)	39 (29.5)
2	8 (6.1)	9 (6.5)
3	2 (1.5)	3 (2.2)
4	0 (0)	1 (0.7)

Mann-Whitney U-test, *p* = 0.866

**Fig. 3:**
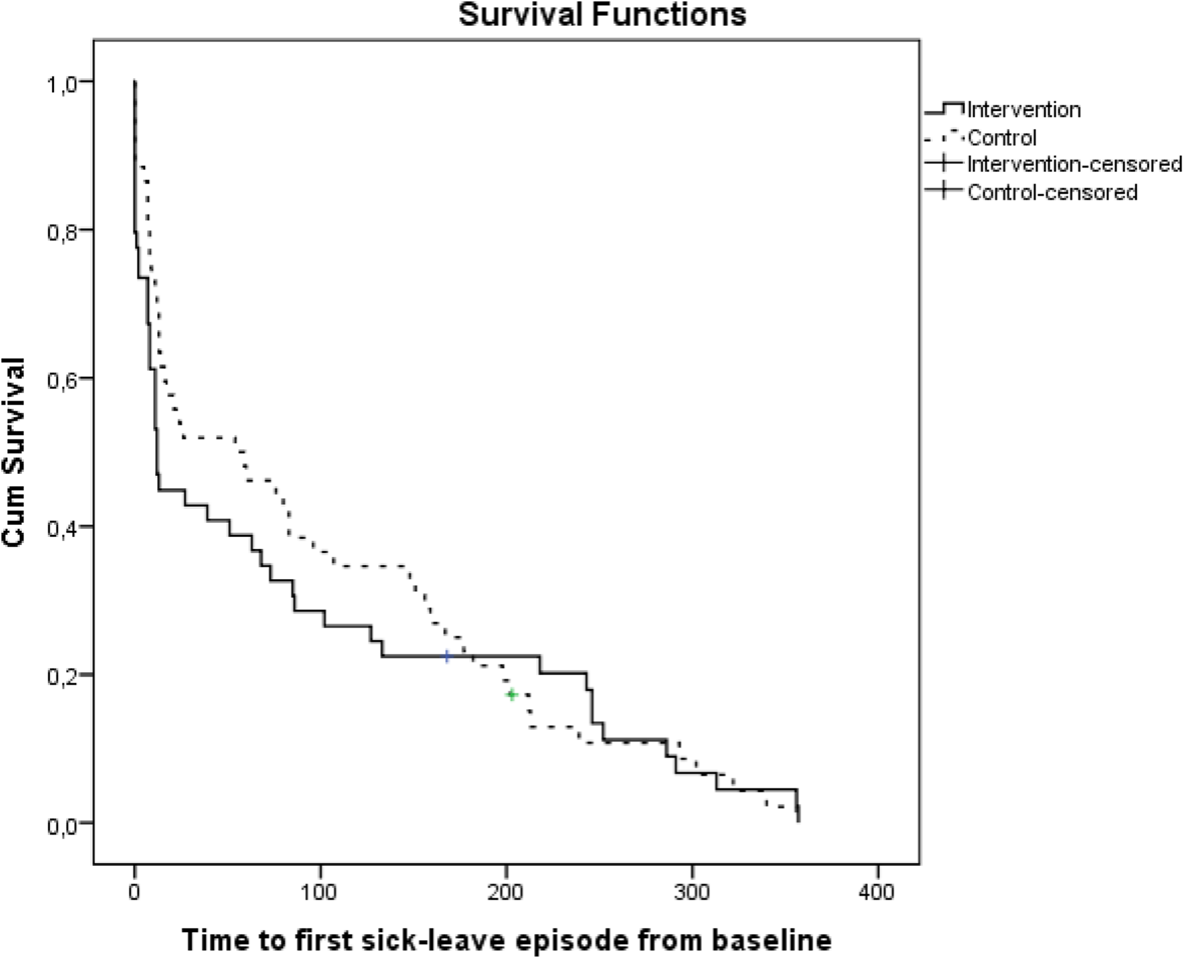
Kaplan- Kaplan-Meier survival curve for time to first SA among those who were sick-listed during the study period, the intervention group (*n* = 49) and control groups (*n* = 52), TIDAS in New Ways

Figure 3 shows the Kaplan-Meier survival curves for time to first day of sick leave episode for the intervention and the control group. Log-rank test showed no significant difference between the groups (χ^2^ = 0.200, df = 1, *p* = 0.655).

## Discussion

In the present study no statistically significant differences were found between the WSQ brief intervention group and the control group, receiving treatment as usual, in the number of registered sick leave days and episodes, or in the time to first day of sick leave episode during the 12-months follow-up. Since there is a lack of measures to support persons suffering from work-related stress and to prevent sick leave and work disability it is important to gain knowledge from different type of interventions to develop future interventions.

### Possible mechanisms and explanations

The main components of the WSQ brief intervention were a brief GP training to raise awareness and knowledge on work-related stress, the completion of the WSQ by participants to promote self-consciousness, feedback on work stress levels at the patient–GP consultation, and finally, good discussions on preventive measures between the GP and the participant. Three out of these four components required the GPs’ active involvement and participation. Although all intervention GPs participated in the training session, no information is available whether the desired effect, raised awareness and knowledge, was reached. Other research found that raising the GPs’ awareness of the risk for future sickness absence due to work-related circumstances increased their knowledge and led them to advice the patient on work-related prevention measures [30, 31]. Another study from the Netherlands on training GP’s to use a minimal intervention for stress-related mental disorders [32], found limited adherence to new routines of GPs after an 11-h training period. In our study, the training session was short as was the intervention period. Training and intervention might have been too short for the participating GPs to get enough knowledge, competence and motivation to provide feedback and discuss preventive measures on issues related to work.

The second component was the WSQ completion, which made it possible for the participants to reflect on their own work situation before the GP-consultation. All participants in the present study completed the WSQ. Earlier research has shown that completion of questionnaires may help patients understand their own problems and improve communication with the GP [33, 34]. To notice, the control group also completed the WSQ, but after the GP-consultation, which might possibly influence their future reasoning and actions in relation to their work place, health care and sick leave.

The third and fourth components involved the GPs whom were trained to actively take part in these intervention components. The third component constitutes the GP feedback on work-related stress based on the WSQ assessment, and information on the risk of future negative outcomes. As the feedback was not standardized, GPs may have provided brief information on the points raised in the WSQ, or more comprehensive information, including the fourth component, by discussing recommendations on relevant measures to reduce work stress or to cope with stress, with the participant. Physician feedback has been shown to be efficient in other brief interventions, in particular when communication techniques such as motivational interviewing were used [35]. A good communication with the GPs seems to be essential for primary health care patients [36], and completing relevant questionnaires prepare the patients for consultation [33, 34]. Earlier studies [34, 37, 38], though, have found a certain resistance among GPs to use instruments, such as the WSQ, which might have contributed to a lack of motivation to fully use the WSQ information.

Another aspect to bear in mind is that the risk for sick leave due to work-related stress is of interest to several other health care professionals and stakeholders. These stakeholders, for example the employer, occupational health services and social insurance agency, might also get involved, and take preventive measures to decrease the risk for sick leave.

### Comparison with relevant findings from previous comparable studies

Kant et al. [39] found that screening for employees at high risk for future long-term sickness absence in combination with early consultation with an occupational physician resulted in reduced sick leave during 12-months follow up compared to treatment as usual. Two important aspects differ between Kant’s study and the present study: the outcome of sick leave and the setting. The outcome in Kant et al. is based on record linkage on an individual level with the company registers on certified sickness absence, which includes short-term sick leave. In the present study, data on short-term sick leave (< 14 days) has not been analyzed, only registered sick leave data on 14 days or more was used. In other words, we do not know if the WSQ brief intervention had an effect on non-registered short-term sick leave. Moreover, the study by Kant et al. [39] took place in the Dutch occupational health services setting engaging occupational physicians, whereas the present study took place in the primary health care engaging GPs. Earlier research has found that although sickness certificates, evaluation of work status and work ability are common GP tasks, little training has been provided [22, 24, 40]. Inadequate knowledge of workplace environment as well as labor market is stated as common by the GPs themselves [22, 24]. Compared to occupational physicians, GPs express a lack of competence in handling issues related to their patients work situation [21, 23]. Osteras et al. (2009) found that a one-day work-shop, including training on functional assessments and patient work factors increased GPs’ knowledge of their patient’s workplace and perceived stressors [31]. In the present study, the intervention GPs training might have been too short to help them gaining knowledge, competency and motivation to counsel the patient to preventive measures.

### Strengths and limitations of the present study

The study was conducted in the primary health context in Sweden, and was designed as a pragmatic RCT. Very few RCTs in primary health care have addressed sick leave, and most have shown no or little impact on sick leave rate [41–45]. Sick leave is a complex phenomenon and not only the result of reduced health. It reflects a combination of personal characteristics, health, work tasks and environment, treatment, vocational measures and the social insurance system [46]. Being a complex phenomenon means that the process is difficult to predict and that measures to prevent sickness absence at the individual level probably must be personalized and combined with short-term follow-up, which is difficult to achieve in structured a RCT.

The randomizing was done at the GP level which might constitute to contamination. The GPs might also discuss the study procedure and how to handle the patients in question among themselves. This risk however was considered as low due to the short inclusion period and brief intervention, which in turn was woven into the ordinary daily practice. Engaging the whole PHCC in recruiting participants to the two groups has been beneficial to the amount of people attending research [27].

The strength of the present study was the use of registered sick leave data from SSIA’s MiDAS, such as the number of registered gross and net sick-days, and the number of sick leave episodes. These measures have also been recommended by SSIA [47]. The advantage of using registered data is that the risk for drop-outs are negligible. Another advantage of registered over self-reported sick leave data is that it eliminates the risk of recall bias. Yet, access to self-reported short-term sick leave data might have given us more precise findings on differences between the groups.

A limitation of the study was that the sick leave data was extensively skewed with most individuals having no registered sick leave (i.e. > 14 days or more) while a few individuals present with very long episodes. Only one third of the participants in this study had registered sick leave. The low rate of individuals on sick leave at follow-up and the extensively skewed population of individuals with registered sick leave, may be the reasons that no statistically significant differences were found, even though there was a 13% difference in mean gross days and, a 20% difference in mean net days between the groups. The standard variation in this study was almost twice the mean at 12 months, which was hard to anticipate since there were few comparable studies published on the subject at the time. Similar problems were found in a study by Arends et al. [48] where recurrent sickness absence days between the two treatment groups were not analyzed due to the skewed distribution and to that more than 50% of the study population had no recurrent sickness absence days at follow-up. The power calculation of our study was based on a previous study [49] that aimed to detect at least 15% difference in proportion for returning to work between the groups, so possibly there was an underestimation of the proportion and variation in length of future sick leave in this study’s population. Hence, the lack of difference between the groups might be due to a type II error. The population of the present study consisted of individuals at risk but not yet sick-listed. To our best knowledge little research on preventing sick leave is available to guide the estimations of a future sick leave level in that specific population.

Another limitation of the study was that we do not know whether the GPs followed all our instructions for the intervention, especially the last two components, or not. That is, we do not know in detail how the GPs provided the participants with the WSQ-information, and how comprehensive the discussion on relevant measures were. We know from earlier research [22, 24, 32] that GPs report little knowledge on the impact of work-related factors on health and sick leave, and that they have few tools for handling work-related issues. Probably, more effort should have been directed to the GPs, to give them enough information on where to refer to other professionals with expert knowledge on these issues. An ongoing process evaluation (Hultén, Dahlin-Ivanoff, Holmgren. GPs’ reasoning about using the Work Stress Questionnaire combined with feedback at consultation in primary health care – A preventive measure for sick-leave due to work-related stress, in preparation) might give us some insight into how the GPs managed the intervention.

Just a few differences were found: age distribution (the proportion of participants aged 51–64 years was larger in the intervention group), and reasons for consultation (the proportion of participants with musculoskeletal disorders was larger in the intervention group) [25]. We have no reason to believe that these differences would influence the results of this study.

There are many difficulties when performing clinical research in primary care, especially randomized controlled trials. There is a need to balance research quality with pragmatism in performance, internal validity and external validity. The possibility for the RCT to be successfully conducted depends on whether it fits into regular care and the health care system. Therefore, the brief intervention had to stay close to regular care as there is a high demand on access and prompt management of different conditions within primary care. However, a brief intervention might just be too brief to be a contrast to treatment as usual.

## Conclusions

The present study did not show that the WSQ brief intervention combined with feedback and suggestions of measures at patient-GP-consultation could prevent sick leave the following 12 months. However, the study may have been underpowered and the lack of difference between the groups might be due to a type II error. We do know that work-related stress constitutes an obvious risk for becoming sick-listed, and that primary health care has no established method to early identify people at risk for sick leave. Hence, more rigorous evaluation studies of the WSQ brief intervention are needed. Also, more research is needed on methods to early identify, advise and treat people with work-related stress in primary health care, and on how and when GPs and other professionals in primary health care can be trained to understand this risk of sick leave due to work-related stress, on how to prevent it, and on how to advise and treat employees at risk.

## Data Availability

The datasets generated and analyzed during the current study are not publicly available due ethical grounds but are available from the corresponding author on reasonable request.

## Abbreviations

CI: Confidence interval
GP: General practitioner
MiDAS: Micro Database for Analyzing Social insurance
PHCC: Primary Health Care Centers
RCT: Randomized controlled trial
SSIA: Swedish Social Insurance Agency
WSQ: Work Stress Questionnaire

## References

[1] Theorell T, Hammarstrom A, Aronsson G, Traskman Bendz L, Grape T, Hogstedt C (2015). A systematic review including meta-analysis of work environment and depressive symptoms. BMC Public Health.

[2] Harvey SB, Modini M, Joyce S, Milligan-Saville JS, Tan L, Mykletun A (2017). Can work make you mentally ill? a systematic meta-review of work-related risk factors for common mental health problems. Occup Environ Med.

[3] Szeto AC, Dobson KS (2013). Mental disorders and their association with perceived work stress: an investigation of the 2010 Canadian community health survey. J Occup Health Psychol.

[4] Hauke A, Flintrop J, Brun E, Rugulies R (2011). The impact of work-related psychosocial stressors on the onset of musculoskeletal disorders in specific body regions: a review and meta-analysis of 54 longitudinal studies. Work Stress.

[5] Wiegner L, Hange D, Bjorkelund C, Ahlborg G (2015). Prevalence of perceived stress and associations to symptoms of exhaustion, depression and anxiety in a working age population seeking primary care--an observational study. BMC Fam Pract.

[6] Holmgren K, Fjallstrom-Lundgren M, Hensing G (2013). Early identification of work-related stress predicted sickness absence in employed women with musculoskeletal or mental disorders: a prospective, longitudinal study in a primary health care setting. Disabil Rehabil.

[7] Mather L, Bergstrom G, Blom V, Svedberg P (2015). High job demands, job strain, and Iso-strain are risk factors for sick leave due to mental disorders: a prospective Swedish twin study with a 5-year follow-up. J Occup Environ Med.

[8] Theorell Töres, Hammarström Anne, Gustafsson Per E, Magnusson Hanson Linda, Janlert Urban, Westerlund Hugo (2013). Job strain and depressive symptoms in men and women: a prospective study of the working population in Sweden. Journal of Epidemiology and Community Health.

[9] Nieuwenhuijsen K, Bruinvels D, Frings-Dresen M (2010). Psychosocial work environment and stress-related disorders, a systematic review. Occup Med (Lond).

[10] Burton C (2003). Beyond somatisation: a review of the understanding and treatment of medically unexplained physical symptoms (MUPS). Br J Gen Pract.

[11] Toft T, Fink P, Oernboel E, Christensen K, Frostholm L, Olesen F (2005). Mental disorders in primary care: prevalence and co-morbidity among disorders. Results from the functional illness in primary care (FIP) study. Psychol Med.

[12] Reid S, Wessely S, Crayford T, Hotopf M (2001). Medically unexplained symptoms in frequent attenders of secondary health care: retrospective cohort study. BMJ.

[13] Holmgren K, Dahlin IS (2004). Women on sickness absence--views of possibilities and obstacles for returning to work. a focus group study. Disabil Rehabil.

[14] Linton SJ, Gross D, Schultz IZ, Main C, Cote P, Pransky G (2005). Prognosis and the identification of workers risking disability: research issues and directions for future research. J Occup Rehabil.

[15] Shaw WS, van der Windt DA, Main CJ, Loisel P, Linton SJ (2009). Early patient screening and intervention to address individual-level occupational factors (“blue flags”) in back disability. J Occup Rehabil.

[16] Linton SJ, Boersma K (2003). Early identification of patients at risk of developing a persistent back problem: the predictive validity of the Orebro musculoskeletal pain questionnaire. Clin J Pain.

[17] Boersma K, Linton SJ (2005). Screening to identify patients at risk: profiles of psychological risk factors for early intervention. Clin J Pain.

[18] Lexis MA, Jansen NW, van Amelsvoort LG, Huibers MJ, Berkouwer A, Tjin ATG (2012). Prediction of long-term sickness absence among employees with depressive complaints. J Occup Rehabil.

[19] Wandell P, Carlsson AC, Wettermark B, Lord G, Cars T, Ljunggren G (2013). Most common diseases diagnosed in primary care in Stockholm, Sweden, in 2011. Fam Pract.

[20] Holmgren K, Hensing G, Dahlin-Ivanoff S (2009). Development of a questionnaire assessing work-related stress in women - identifying individuals who risk being put on sick leave. Disabil Rehabil.

[21] Buijs PC, van Dijk FJ, Evers M, vd Klink JJ, Anema H (2007). Managing work-related psychological complaints by general practitioners, in coordination with occupational physicians: a pilot study. Ind Health.

[22] Nilsing Emma, Söderberg Elsy, Berterö Carina, Öberg Birgitta (2013). Primary Healthcare Professionals’ Experiences of the Sick Leave Process: A Focus Group Study in Sweden. Journal of Occupational Rehabilitation.

[23] Anema JR, Jettinghoff K, Houtman I, Schoemaker CG, Buijs PC, van den Berg R (2006). Medical care of employees long-term sick listed due to mental health problems: a cohort study to describe and compare the care of the occupational physician and the general practitioner. J Occup Rehabil.

[24] Pransky G, Katz JN, Benjamin K, Himmelstein J (2002). Improving the physician role in evaluating work ability and managing disability: a survey of primary care practitioners. Disabil Rehabil.

[25] Holmgren K, Sandheimer C, Mardby AC, Larsson ME, Bultmann U, Hange D (2016). Early identification in primary health care of people at risk for sick leave due to work-related stress - study protocol of a randomized controlled trial (RCT). BMC Public Health.

[26] Zwarenstein M, Treweek S, Gagnier JJ, Altman DG, Tunis S, Haynes B (2008). Improving the reporting of pragmatic trials: an extension of the CONSORT statement. BMJ.

[27] Mortenius H, Baigi A, Palm L, Fridlund B, Bjorkelund C, Hedberg B (2015). Impact of the organisational culture on primary care staff members' intention to engage in research and development. J Health Organ Manag.

[28] Regions SAoLAa. [In Rehabiliteringsgarantins effekter på hälsa och sjukfrånvaro Rehabiliteringsgarantin 2014 [In English: The effects of the Swedish rehabilitation warranty on health and sickness absence. The rehabilitation warranty 2014]. Available at https://skl.se/halsasjukvard/sjukskrivningochrehabilitering/smartaochpsykiskohalsa/utvarderingrehabiliteringsgarantin. 2014.

[29] Altman D (1999). Practical statistics for medical research.

[30] Verger P, Menard C, Richard JB, Demortiere G, Beck F (2014). Collaboration between general practitioners and occupational physicians: a comparison of the results of two national surveys in France. J Occup Environ Med.

[31] Osteras N, Gulbrandsen P, Benth JS, Hofoss D, Brage S (2009). Implementing structured functional assessments in general practice for persons with long-term sick leave: a cluster randomised controlled trial. BMC Fam Pract.

[32] Bakker IM, van Marwijk HW, Terluin B, Anema JR, van Mechelen W, Stalman WA (2010). Training GP’s to use a minimal intervention for stress-related mental disorders with sick leave (MISS): effects on performance: results of the MISS project; a cluster-randomised controlled trial. Patient Educ Couns.

[33] Wikberg C, Pettersson A, Westman J, Bjorkelund C, Petersson EL (2016). Patients’ perspectives on the use of the Montgomery-Asberg depression rating scale self-assessment version in primary care. Scand J Prim Health Care.

[34] Dowrick C, Leydon GM, McBride A, Howe A, Burgess H, Clarke P (2009). Patients’ and doctors’ views on depression severity questionnaires incentivised in UK quality and outcomes framework: qualitative study. BMJ.

[35] Rosario F, Santos MI, Angus K, Pas L, Fitzgerald N (2018). Factors influencing the implementation of screening and brief interventions for alcohol use in primary care practices: a systematic review protocol. Acta Medica Port.

[36] Paddison CA, Abel GA, Roland MO, Elliott MN, Lyratzopoulos G, Campbell JL (2015). Drivers of overall satisfaction with primary care: evidence from the English general practice patient survey. Health Expect.

[37] Pettersson A, Bjorkelund C, Petersson EL (2014). To score or not to score: a qualitative study on GPs views on the use of instruments for depression. Fam Pract.

[38] Schumann I, Schneider A, Kantert C, Lowe B, Linde K (2012). Physicians’ attitudes, diagnostic process and barriers regarding depression diagnosis in primary care: a systematic review of qualitative studies. Fam Pract.

[39] Kant I, Jansen NW, van Amelsvoort LG, van Leusden R, Berkouwer A (2008). Structured early consultation with the occupational physician reduces sickness absence among office workers at high risk for long-term sickness absence: a randomized controlled trial. J Occup Rehabil.

[40] Lofgren A, Hagberg J, Arrelov B, Ponzer S, Alexanderson K (2007). Frequency and nature of problems associated with sickness certification tasks: a cross-sectional questionnaire study of 5455 physicians. Scand J Prim Health Care.

[41] Wikberg C, Westman J, Petersson EL, Larsson ME, Andre M, Eggertsen R (2017). Use of a self-rating scale to monitor depression severity in recurrent GP consultations in primary care - does it really make a difference? a randomised controlled study. BMC Fam Pract.

[42] Hange D, Ariai N, Kivi M, Eriksson MC, Nejati S, Petersson EL (2017). The impact of internet-based cognitive behavior therapy on work ability in patients with depression - a randomized controlled study. Int J Gen Med.

[43] Bjorkelund C, Svenningsson I, Hange D, Udo C, Petersson EL, Ariai N (2018). Clinical effectiveness of care managers in collaborative care for patients with depression in Swedish primary health care: a pragmatic cluster randomized controlled trial. BMC Fam Pract.

[44] Bakker IM, Terluin B, van Marwijk HW, van der Windt DA, Rijmen F, van Mechelen W (2007). A cluster-randomised trial evaluating an intervention for patients with stress-related mental disorders and sick leave in primary care. PLoS Clin Trials.

[45] Huibers MJ, Beurskens AJ, Van Schayck CP, Bazelmans E, Metsemakers JF, Knottnerus JA (2004). Efficacy of cognitive-behavioural therapy by general practitioners for unexplained fatigue among employees: randomised controlled trial. Br J Psychiatry.

[46] Hensing Gunnel (2004). Chapter 4. Methodological aspects in sickness-absence research. Scandinavian Journal of Public Health.

[47] Försäkringskassan (The Swedish Social Insurance Agency) (2016). Förslag på utfallsmått för att mäta återgång i arbete efter sjukskrivning (Suggestions for outcome criteria for measuring return to work after sick leave).

[48] Arends I, van der Klink JJ, van Rhenen W, de Boer MR, Bultmann U (2014). Prevention of recurrent sickness absence in workers with common mental disorders: results of a cluster-randomised controlled trial. Occup Environ Med.

[49] van Beurden KM, Brouwers EP, Joosen MC, Terluin B, van der Klink JJ, van Weeghel J (2013). Effectiveness of guideline-based care by occupational physicians on the return-to-work of workers with common mental disorders: design of a cluster-randomised controlled trial. BMC Public Health.

